# Effects of cadmium sulfide nanoparticles on sulfate bioreduction and oxidative stress in *Desulfovibrio desulfuricans*

**DOI:** 10.1186/s40643-022-00523-5

**Published:** 2022-04-01

**Authors:** Guoqing Cheng, Huili Ding, Guanglin Chen, Hongjie Shi, Xu Zhang, Minglong Zhu, Wensong Tan

**Affiliations:** State Key Laboratory of Bioreactor Engineering, East China University of Science and Technology, Shanghai, 200237 China

**Keywords:** *Desulfovibrio desulfuricans*, Cadmium sulfide nanoparticles, Sulfate reduction, Extracellular polymeric substances (EPS), Oxidative stress

## Abstract

Sulfate-containing wastewater has a serious threat to the environment and human health. Microbial technology has great potential for the treatment of sulfate-containing wastewater. It was found that nano-photocatalysts could be used as extracellular electron donors to promote the growth and metabolic activity of non-photosynthetic microorganisms. However, nano-photocatalysts could also induce oxidative stress and damage cells. Therefore, the interaction mechanism between photosynthetic nanocatalysts and non-photosynthetic microorganisms is crucial to determine the regulatory strategies for microbial wastewater treatment technologies. In this paper, the mechanism and regulation strategy of cadmium sulfide nanoparticles (CdS NPs) on the growth of sulfate-reducing bacteria and the sulfate reduction process were investigated. The results showed that the sulfate reduction efficiency could be increased by 6.4% through CdS NPs under light conditions. However, the growth of *Desulfovibrio desulfuricans* C09 was seriously inhibited by 55% due to the oxidative stress induced by CdS NPs on cells. The biomass and sulfate reduction efficiency could be enhanced by 6.8% and 5.9%, respectively, through external addition of humic acid (HA). At the same time, the mechanism of the CdS NPs strengthening the sulfate reduction process by sulfate bacteria was also studied which can provide important theoretical guidance and technical support for the development of microbial technology combined with extracellular electron transfer (EET) for the treatment of sulfate-containing wastewater.

**Graphical Abstract:**

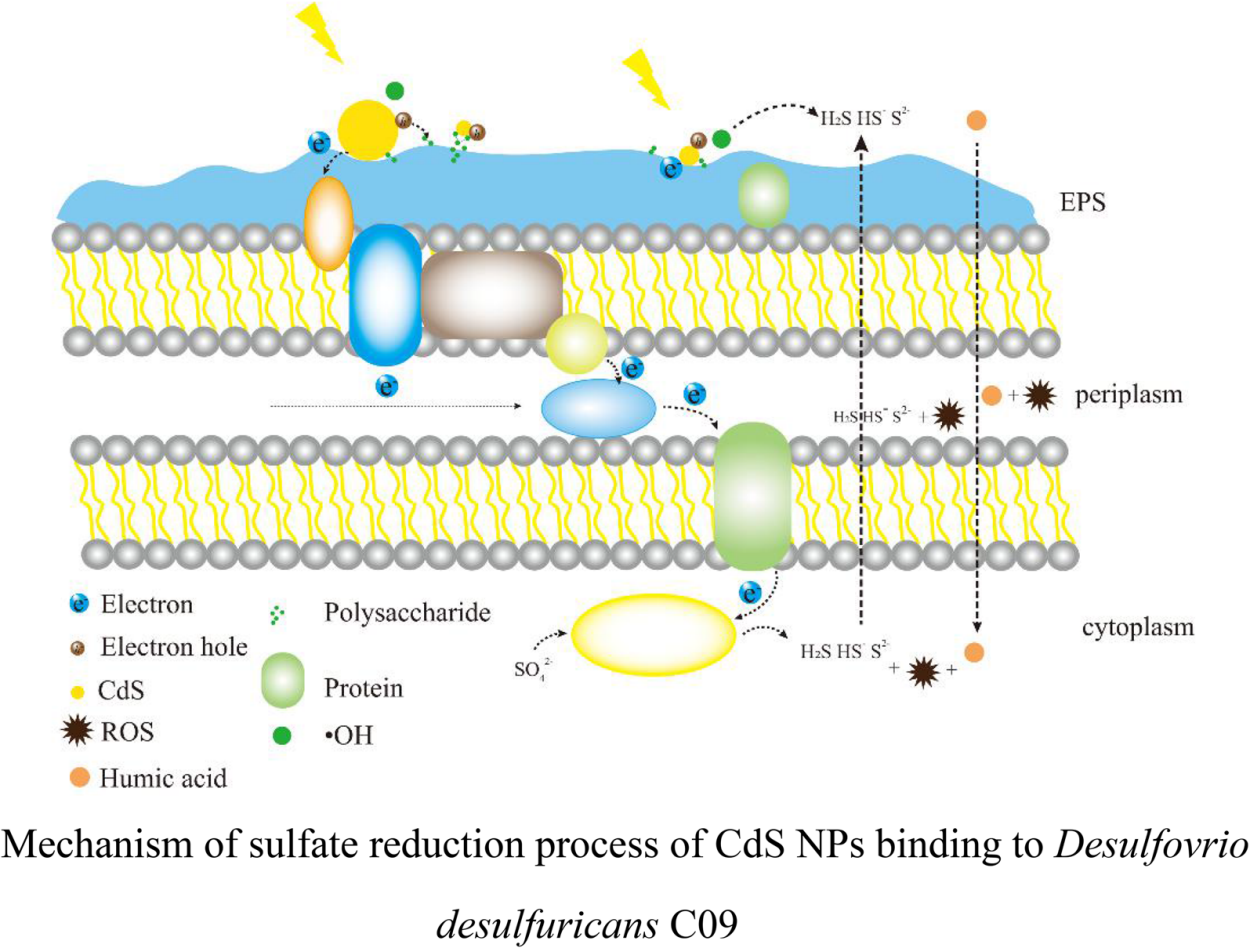

**Supplementary Information:**

The online version contains supplementary material available at 10.1186/s40643-022-00523-5.

## Highlight

- Efficiency of bioreduction of sulfate and biomass can be increased by CdS NPs.
- It is important for the EPS of RSB to improve the sulfate reduction efficiency.
- Utilization efficiency of extracellular electrons by RSB can be enhanced through EPS.
- Humic acid can alleviate the oxidative stress induced by CdS NPs to SRB.

**Supplementary Information:**

The online version contains supplementary material available at 10.1186/s40643-022-00523-5.

## Introduction

The discharge of industrial wastewater containing sulfate have been causing an imbalance in natural microbiota (algae, bacteria and fungi) of water ecosystems and soil, which not only pollutes the natural environment, but also affects human health (Radić et al. 2014). Chemical, physical, and biological techniques are commonly used in the treatment of sulfate-containing wastewater. Among them, biotechnology is considered to be the most promising method. Commonly, it mainly used sulfate-reducing bacteria to reduce sulfate to S^2−^ through dissimilatory action. The carbohydrate is the electron donor, and the sulfate ion is the electron acceptor (Peck et al. 1982; Deng et al. 2018). Research on the development of applied bioreactors had been carried out for this technology (Liu et al. 2018; Melgao et al. 2020).

It was found that microorganisms could carry out extracellular respiration energy metabolism (Lovley et al. 2008), which could consume the extracellular electron to promote the growth of microbial and the transference of energy (Xiao et al. 2017). Microbial could achieve extracellular electron transfer (EET) through the interaction of cytochromes, electronic shuttles, and others conductive materials in the out membrane (Li et al. 2012, 2019). Previous studies have found that extracellular polymeric substances (EPS) could play a very important role in the process of EET (Xiao et al. 2017; Sathishkumar et al. 2020). For example, results of proteomic analysis of *Shewanella* s*oneidensis* HRCR-1 showed that there was a large number of proteins in extracellular polymeric substances (EPS), among which c-type cytochromes and riboflavin redox substances MtrC and OmcA were related to EET (Cao et al. 2011). The EPS of *S. oneidensis* MR-1 included cytochromes and riboflavin redox substances (Xiao et al. 2017). The EPS of *S. oneidensis* and *Pseudomonas putida* contained heme-binding protein related to EET (Li et al. 2016). The quinone in humic acid could act as an electron shuttle to accelerate the reduction of dissimilated ferric ion (Wolf et al. 2009). Therefore, some substances in EPS could mediate EET of microorganisms.

As a photocatalyst, CdS NPs had the characteristics of low band gap and high photosensitivity, which could generate photo-excited electrons under light conditions (Prabhu et al. 2005). It was found that these electrons could be consumed to enhance certain metabolic capabilities of microbial (Dong et al. 2020). The biohydrogen production of *Escherichia coil* was also increased when the cell *s*urface precipitated CdS NPs under visible light illumination (Wang et al. 2017). CdS NPs could provide photo-excited electrons for *Rhodopseudomonas palustris* to enhance nitrate reduction. In addition, CdS NPs could be excited to generate photoelectrons and holes under light conditions. Then cysteine was used as an electron sacrificial agent, and the electrons combine with H^+^ to form [H] for *Moorella thermoacetica* to reduce CO_2_ to acetic acid (Gai et al. 2020). Enhanced reduction efficiency of sulfate under sulfate-reducing condition was obtained by synergism between EPS and graphene oxide (Yan et al. 2020). Therefore, the photosensitive nanomaterials could indeed provide energy for bacteria in the form of EET.

However, it was found that the hydroxyl free radicals could be generated by oxidizing water by CdS NPs under light conditions. The increased intracellular reactive oxygen species (ROS) content will cause the oxidative stress effect in the microbial cells. The oxidative stress effect of CdS NPs on *E. coli* (ATCC 13,534), *E.coli* (ATCC 25,922) and *Staphylococcus aureus* (ATCC 25,923) were investigated, respectively. It was found that more than 90% growth of biomass were severely inhibited when the cells were treated with CdS NPs of 1 μg/mL (Chandran et al. 2014). The activity of *Bacillus subtilis* and *E. coli* could be inhibited by NiO NPs due to the damage of ROS produced by NiO NPs (Behera et al. 2019). Fortunately, it has been found that many natural organic macromolecular substances could alleviate the damage of oxidative stress to microorganisms, such as humic acid (HA). It was shown that HA could reduce the distribution of CdS NPs in solution, thereby decrease the amount of the production of ·OH and ^1^O_2_ (Shang et al. 2017a, b). The photocatalytic activity of graphene–cadmium sulfide (G–CdS) nanocomposites on the bactericidal effect of *E. coli* was studied. It was demonstrated that the addition of HA could react with ROS to greatly reduce the toxicity of G–CdS to *E. coli* (Deng et al. 2016). In summary, the external addition of HA could be used as an ROS scavenger to reduce the amount of ROS generated by nanoparticles.

On the one hand, some photo-nano-catalysts can act as extracellular electron donors to promote the growth and metabolic activity of non-photosynthetic microorganisms. On the other hand, photo-nano-catalysts can also produce cytotoxicity, such as generating oxidative free radicals, which can induce oxidative stress and damage cells. Similar problems still existed in the process using CdS NPs to promote the activity of sulfate-reducing bacteria (SRB) to treat sulfate-containing wastewater, but there were no detailed public reports.

In this paper, a self-screened SRB combined with the self-synthesized CdS NPs were adopted to explore the process mechanism of sulfate reduction by sulfate-reducing bacteria strengthened by CdS NPs. Moreover, the effect of oxidative stress on SRB induced by CdS NPs and the corresponding control strategies were also studied. The results can provide important theoretical guidance and technical support for the development of microbial technology combined with EET for the treatment of sulfate-containing wastewater.

## Materials and methods

### Strain isolation and identification

Sulfate reducing bacteria (SRB) was isolated from river sludge, Shanghai, China. The sludge samples containing SRBs were collected in sealing bag and stored at 4℃. The bacteria were enriched in medium containing Fe^2+^ and isolated under anaerobic incubator at 35℃. It is mainly because Fe^2+^ can react with S^2−^ from SO_4_^2−^ bioreduction reaction to form black FeS precipitate, so this phenomenon can be used as an indicator.

The characteristics of SRB were investigated.

DNA extraction and 16S rRNA sequencing were carried out by Beijing Liuhe Bgi Co. Ltd. The 16S rRNA gene sequences of the type strains of the various genera used in this study were retrieved from the NCBI database and used for cladistic analysis. Sequences with more than 99% similarity were selected for analysis. Phylogenetic trees were constructed using the neighbor-joining method in MEGA program version 4.1. According to above results, the target strain was identified as genus *Desulfovibrio* and named as *D. desulfuricans* C09.

The results about the screening and identification of sulfate-reducing bacteria could be seen in supplementary material.

### Cultured medium and condition

The SRB was cultured using the Postgate medium. It was (pH 7.0 ± 0.2) consisted of 0.5 g/L K_2_HPO_4_, 1.0 g/L NH_4_Cl, 0.1 g/L CaCl_2_·2H_2_O, 2.0 g/L MgSO_4_·7H_2_O, 2.0 g/L (60% w/v) DL-Na-lactate, 1.0 g/L yeast extract, 0.1 g/L ascorbic acid, 0.2 g/L (NH_4_)_2_Fe(SO_4_)_2_·6H_2_O, which were obtained from Shanghai Aladdin Reagent Company (Shanghai, China). All chemical were of analytical reagent grade.

The isolated strain was incubated in the sterile medium and cultured in a 250-mL serum bottle with working volume of 100 mL at 35℃ at 100 rpm in a shaker.

To provide anaerobic condition, serum bottles were flushed with N_2_ (99.99%) for at least 20 min. Anaerobic indicator (resazurin) was purchased from ThermoFisher. When the solution was oxygen-free, the color of the solution changed from pink to colorless.

### Synthesis of cadmium sulfide nanoparticles

The CdS NPs were prepared via the hydrothermal synthesis method reported with some modification (Aboulaich et al. 2012). It was mainly reflected in the change of reaction temperature and time.

The CdCl_2_·2.5H_2_O aqueous solution of 0.175 mM was prepared (about 0.039 g cadmium chloride hydrate in 20 mL aqueous solution). The pH of solution was adjusted to about 10 through NaOH aqueous solution. The thiourea aqueous solution of 0.289 mM was prepared (about 0.022 g thiourea in 20 mL aqueous solution). Then cadmium chloride solution and thiourea solution were mixed and stirred for 20 min. The Na_2_S·9H_2_O aqueous solution of 0.634 mM (about 0.152 g Na_2_S in 20 mL aqueous solution) was slowly dropped into the aforementioned solution under the continuous stirring at room temperature for 30 min. The mixture was transferred to a 100 mL Teflon-line stainless steel autoclave, which was sealed and placed in a 160℃ oven for 8 h. After the reaction, the autoclave was cooled to room temperature naturally. Finally, the yellow precipitate was collected by high-speed centrifugation from the reaction solution. The precipitate was rinsed three times with ethanol and distilled water, and then freeze-dried for use.

### Characterization of cadmium sulfide nanoparticles

The absorption spectrum of CdS was measured using a UV–vis, Spectrophotometer (Varian, USA) in the wavelength range of 200–800 nm.

The crystal structure of the CdS was characterized by X-ray diffraction (Rigaku, Japan) at voltage of 40 kV using monochromatic CuKα radiation. The microstructure of the CdS NPs was characterized by scanning electron microscope (SEM, Nova NanoSEM 450, USA).

**Fig. 1:**
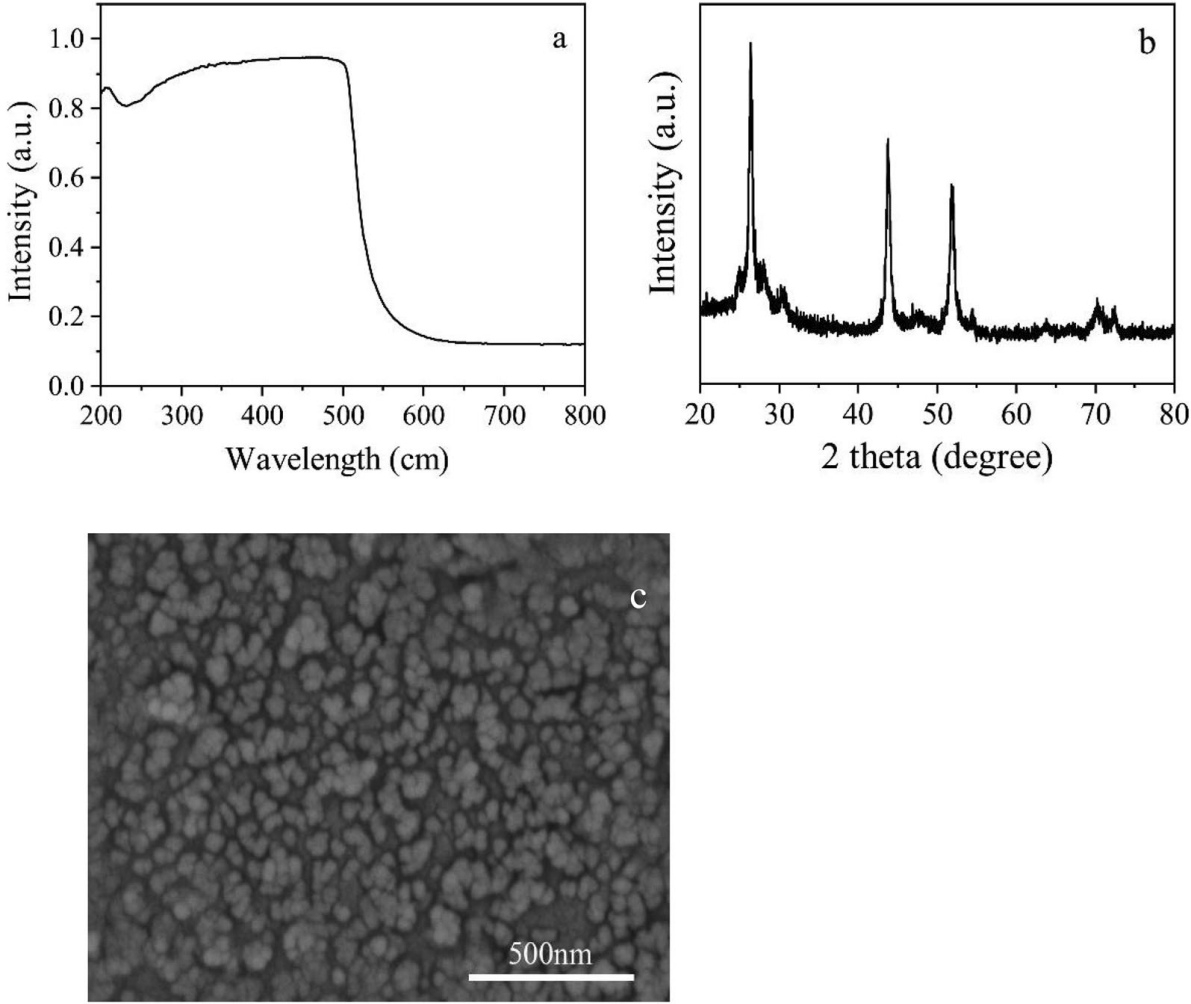
The characteristic of CdS NPs (**a** UV of CdS NPs; **b** XRD of CdS NPs; **c** SEM of CdS NPs)

The UV absorption spectra, XRD, and SEM of CdS NPs are shown in Fig. 1a–c, respectively.

As shown in Fig. 1a, the UV absorption wavelength of CdS NPs was scanned in the range of 200 nm-800 nm. The absorption wavelengths were in the range of 200 nm-600 nm. The maximum absorption wavelength was 506 nm.

1 $${\text{Eg}}\, = \,{124}0/\lambda {\text{g,}}$$

where Eg is the energy band gap value of photocatalyst, (eV); λg is the threshold for absorption wavelength, nm.

According to the formula (1), the band gap of CdS NPs was calculated as 2.4 eV, indicating that CdS NPs had a low band gap (Alipour et al. 2021). It also had absorption characteristics in both the ultraviolet and visible regions.

The XRD pattern of CdS NPs is shown in Fig. 1b. The characteristic diffraction peaks of CdS NPs appeared at 2 theta of 26.5°, 43.3°, and 52.1°, respectively. They were sharp and independent of each other, indicating the high purity and cubic crystals of the CdS NPs synthesized by hydrothermal method (Riaz et al. 2020), which had better photocatalytic effect than the hexagonal crystal system (Lang et al. 2014). The SEM photograph of CdS NPs is shown in in Fig. 1c. It could be seen that they were mainly spherical with the size between 50 and 150 nm.

### Sulfate and cell concentration

The sulfate concentration was measured by the barium sulfate turbidimetric method. One milliliter of culture solution was centrifuged at 12,000 rpm for 10 min, and 0.5 mL of supernatant was taken in a test tube. Adding 0.5 mL of distilled water, 1 mL of 0.5 M hydrochloric acid, 2 mL of polyvinyl alcohol solution, and 2 mL of saturated barium chloride solution into the tube. After mixing, the absorbance of the sample at 420 nm was determined by an ultraviolet–visible spectrophotometer in a 1 cm cuvette (UV-2102C, Unico Shanghai Instruments Co., Ltd., Shanghai, China) in three minutes later. Microscopic counting method was used to determine the amount of bacteria.

### Extracellular polymeric substances extraction, purification and identification

The C09 strain after growth for 24 h was collected by centrifugation at 8000 rpm for 10 min for EPS extraction. Thermal extraction method was used to extract EPS of C09 strain (Dai et al. 2016). The dissolved structural EPS was dialyzed for 24 h against demineralized water in dialysis tubing with a molecular weight cut off 3500 Da, frozen at -80℃ and freeze-dried. The EPS was used for quantification and identification. Three-dimensional excitation emission matrix (EEM) fluorescence spectroscopy (Perkin Elmer, USA) and Fourier transform infrared spectroscopy (FT-IR, Nicolet, USA) was employed to identify EPS. The liquid EPS was used for three-dimensional EEM analysis. The EPS of freeze-dried were mixed with KBr in a wave number range of 4000–500 cm^−1^ for FT-IR analysis. In order to compare the effects of EPS components on the instantaneous photo-current of CdS NPs, EPS was treated with protease (2.5 μL/mL, Proserpina K 10 mg/mL) and polysaccharase (10.0 μL/mL, pullulanase 1000 U/mL) (Yan et al. 2020).

### Analysis of photo-current

The photo-current responses of CdS NPs in the presence or absence of EPS were determined using amperometric I–t curves for 300 s in a three-electrode chamber containing a working electrode (ITO conductive glass, 1 × 1 cm^2^), a counter electrode (a platinum-wire electrode), and a reference electrode (Ag/AgCl electrode with saturated KCl solution). 0.001 g CdS NPs and 20 μL Nafion aqueous solution were mixed in 2 mL ethanol solution and 0.1 M Na_2_SO_4_ aqueous solution. The photo-current change was determined by 0.3 V constant potential. During the test, LED light was used as the light source.

### Reactive oxygen species (ROS), ·OH and malondialdehyde (MDA) content

The bacterial intracellular ROS content was detected using an ROS assay Kit. The cells that had been cultured for 8 h were collected by centrifugation at 8000 rpm for 8 min. And then the pellet was rinsed three times with 0.1 M phosphate balanced solution (PBS), and resuspended in 1 mL 0.1 M PBS to determine OD600. One microliter of 2′,7′-dichlorofluorescin diacetate (DCFH-DA) probe was then added to 1 mL of the cell resuspension solution. After that, it was kept away from light at 37℃ for 20 min in a water bath incubator. After centrifugation at 16,000*g* for 8 min, the cell pellet was washed with 1 mL of sterile water. This washing and centrifuging process was repeated for three times. The cell pellet was resuspended again in 200 µL of sterile water, shaken well, and then transferred to a non-transparent 96-well plate. Then, it was read under a micro-plate reader with excitation and emission wavelengths of 488 and 525 nm, respectively. The fluorescence (FL) value is positively correlated with the ROS content. OD600 was used to represent the amount of cells in a resuspension solution. The ratio of the FL value to the OD600 (FL/OD600) was defined as the intracellular ROS content per unit cell.

The bacterial extracellular ·OH content was detected using an ·OH assay Kit (Nanjing Jiancheng, China) according to the manufacturer’s instructions. The content of MDA was measured from the productions of MDA and thiobarbituric acid (TBA) following Draper (Draper et al. 1990). All test was run in triplicate.

## Results and discussion

### Effect of cadmium sulfide nanoparticles on growth and sulfate reduction process of C09 strain

**Fig. 2:**
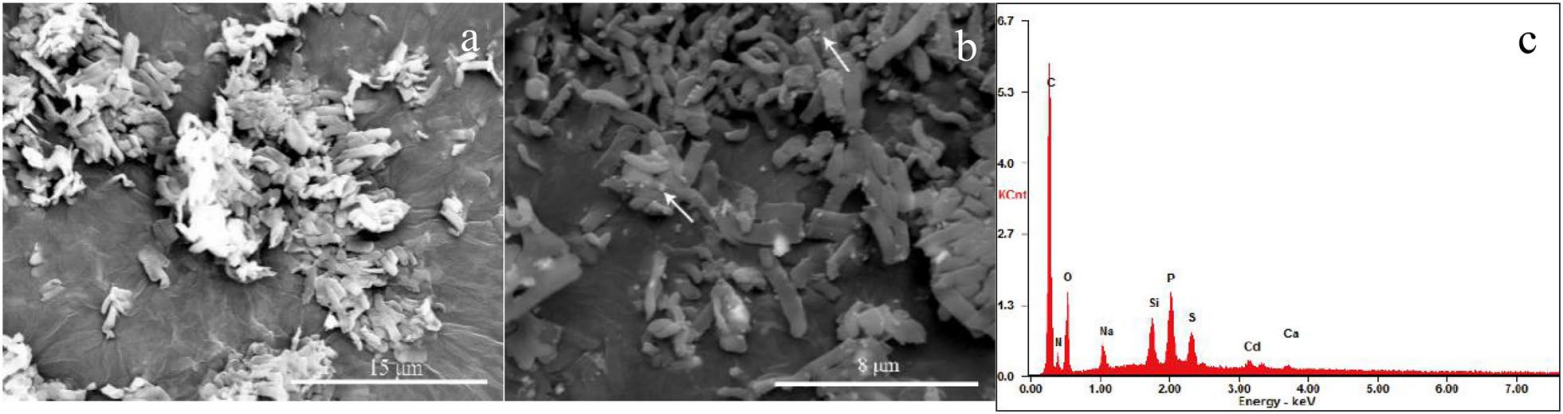
The SEM image and its corresponding EDS spectrum of C09 after treatment with CdS NPs. **a** Without CdS NPs;** b** with CdS NPs;** c** EDS of C09 with CdS NPs

When CdS NPs were combined with C09 strain, the surface of C09 strain was studied by scanning electron microscopy (SEM) and energy dispersive spectrometer (EDS) with results shown in Fig. 2.

Figure 2a and b are the images of the cells combined without and with CdS NPs, respectively. It could be clearly seen that a large number of solid particles were attached to the surface of the C09 strain (Fig. 2b). Analysis results by EDS scanning revealed that Cd and S were distributed on the surface of the bacteria (Fig. 2c). Therefore, it could be determined that there were CdS NPs attached to the surface of the C09 strain.

**Fig. 3:**
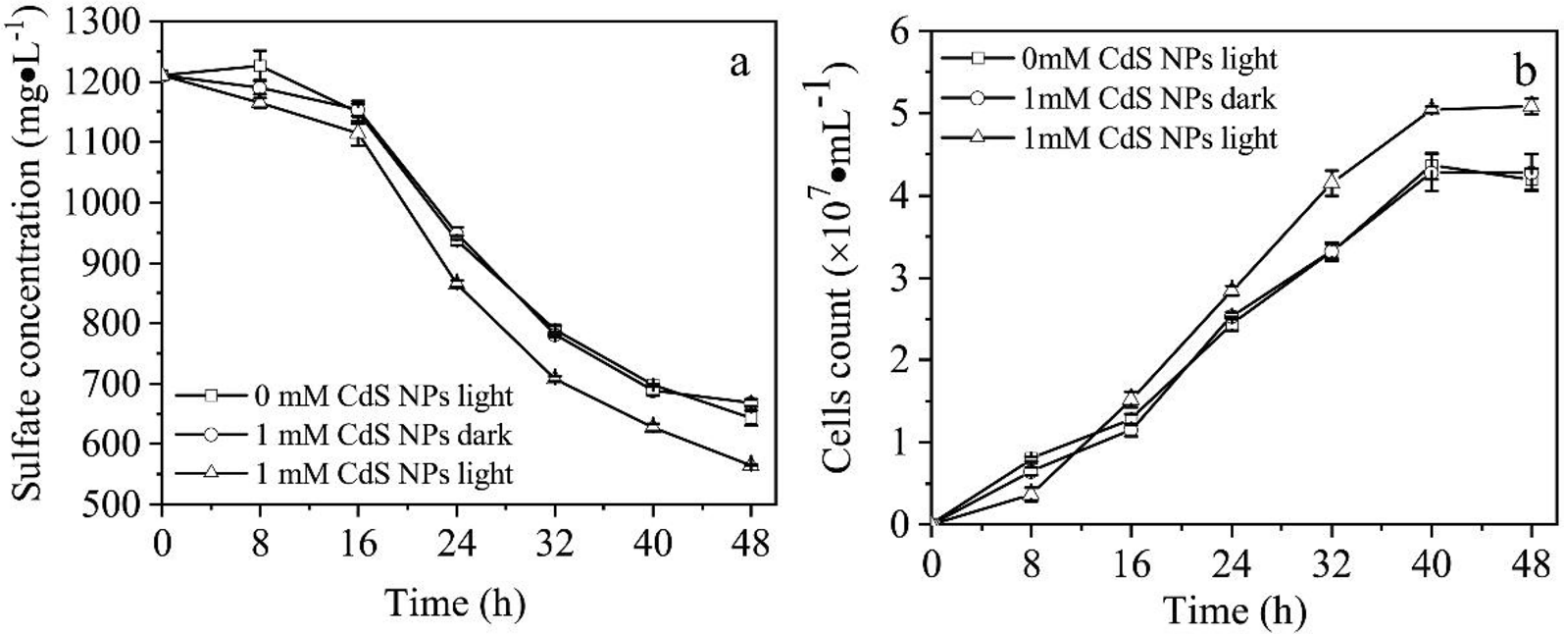
Effects of the CdS NPs on C09 sulfate reduction and growth process.** a** Sulfate concentration;** b** Biomass

The effects of 1 mM CdS NPs on the activities of growth and sulfate reduction of C09 strain were explored in this study with results shown in Fig. 3. It was observed that the sulfate concentration decreased with time due to the sulfate reduction of C09 strain. The sulfate reduction efficiencies of the groups without CdS NPs and with 1 mM CdS NPs in dark were similar because of no light-excited electron generation from CdS NPs. However, the efficiencies of the group without CdS NPs and with 1 mM CdS NPs under light conditions were 46.9% and 53.3%, respectively (Fig. 3a), which means that the reduction efficiency of C09 strain to sulfate could indeed be improved by CdS NPs under light conditions.

In addition, the biomass increased with time under different conditions with results shown in Fig. 3b. Under light conditions, the biomass of the group with 1 mM CdS NPs was significantly higher than that of the other groups, while the amounts of biomass were similar between the groups with CdS NPs under dark conditions and without CdS NPs (Fig. 3b). This means that the growth of C09 strain could not be affected by CdS NPs in dark conditions. The effects of CdS NPs on the nitrate reduction efficiency of attached microorganisms under light conditions were investigated with the results showing that the addition of CdS NPs increased the removal of nitrate by 1.5 times (Zhu et al. 2018). The CdS NPs were adsorbed on the surface of the light-energy heterotrophic bacterium *Rhodopseudomonas palustris* which could not only enhance the nitrogen fixation capacity of the bacteria, but also increase the biomass under light conditions (Wang et al. 2019). Therefore, the growth and sulfate reduction efficiency of C09 strain could indeed be improved by CdS NPs under light conditions.

### Effects of extracellular polymeric substances on the enhanced activity of C09 strain in cadmium sulfide nanoparticles

It was found that microbial EPS could play a very important role in the process of CdS NPs to enhance bacterial metabolism and growth activity. Therefore, this study investigated the influence of CdS NPs on the growth of C09 strain and the sulfate reduction process in the presence or absence of EPS.

To demonstrate the effect of CdS NPs on the growth and reducing capacity of C09 strain in the absence of EPS, the experimental group without EPS (R-EPS) were divided into two groups (added with and without 1 mM CdS NPs). Similarly, to demonstrate the effect of CdS NPs on the growth and reducing capacity of C09 strain in the presence of EPS, the control group with EPS (C-EPS) were also divided into two groups (added with and without 1 mM CdS NPs).

**Fig. 4:**
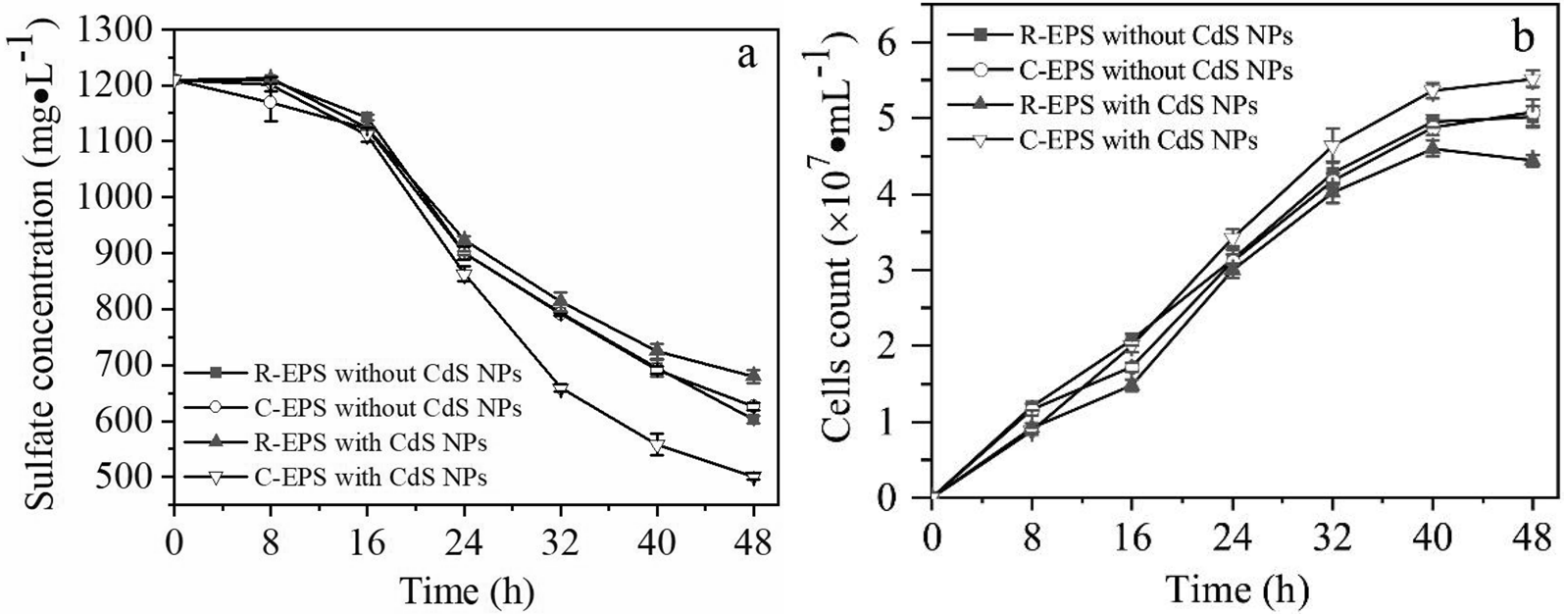
Effects of CdS NPs on sulfate reduction, growth and EPS composition of strain. C09 that removed/contained EPS.** a** Sulfate concentration;** b** Biomass

Regardless of whether CdS NPs were added, the sulfate concentration of all the groups decreased with time, as shown in Fig. 4a. The sulfate reduction efficiencies of the C-EPS group and R-EPS group added with CdS NPs were 58.5% and 43.8%, respectively, while the sulfate reduction efficiencies of the C-EPS group and R-EPS group without CdS NPs were 50.2% and 48.3%, respectively, as shown in Fig. 4a. This result shows that the experimental groups with EPS were retained, and the presence of CdS NPs caused the biomass of C09 strain to be significantly higher than that of the other groups shown in Fig. 4b.

It was well known that most of microorganisms were wrapped by EPS, which contains polysaccharides, proteins, HA, and other substances (Flemming et al. 2007). EPS could not only serve as a space barrier to protect microbial cells, but also participate in electron transfer during cell metabolism. It was found that the reduction of nitrate was inhibited after the removal of EPS (Zhu et al. 2018). Thus, when CdS NPs were added, the sulfate reduction rate and the biomass of C09 strain without extracellular EPS were lower than other groups, which indicating that EPS may mediate the transfer of electrons produced by CdS NPs to C09 strain to participate in sulfate reduction and bacterial growth process.

### The influence of cadmium sulfide nanoparticles on extracellular polymeric substances components

**Fig. 5:**
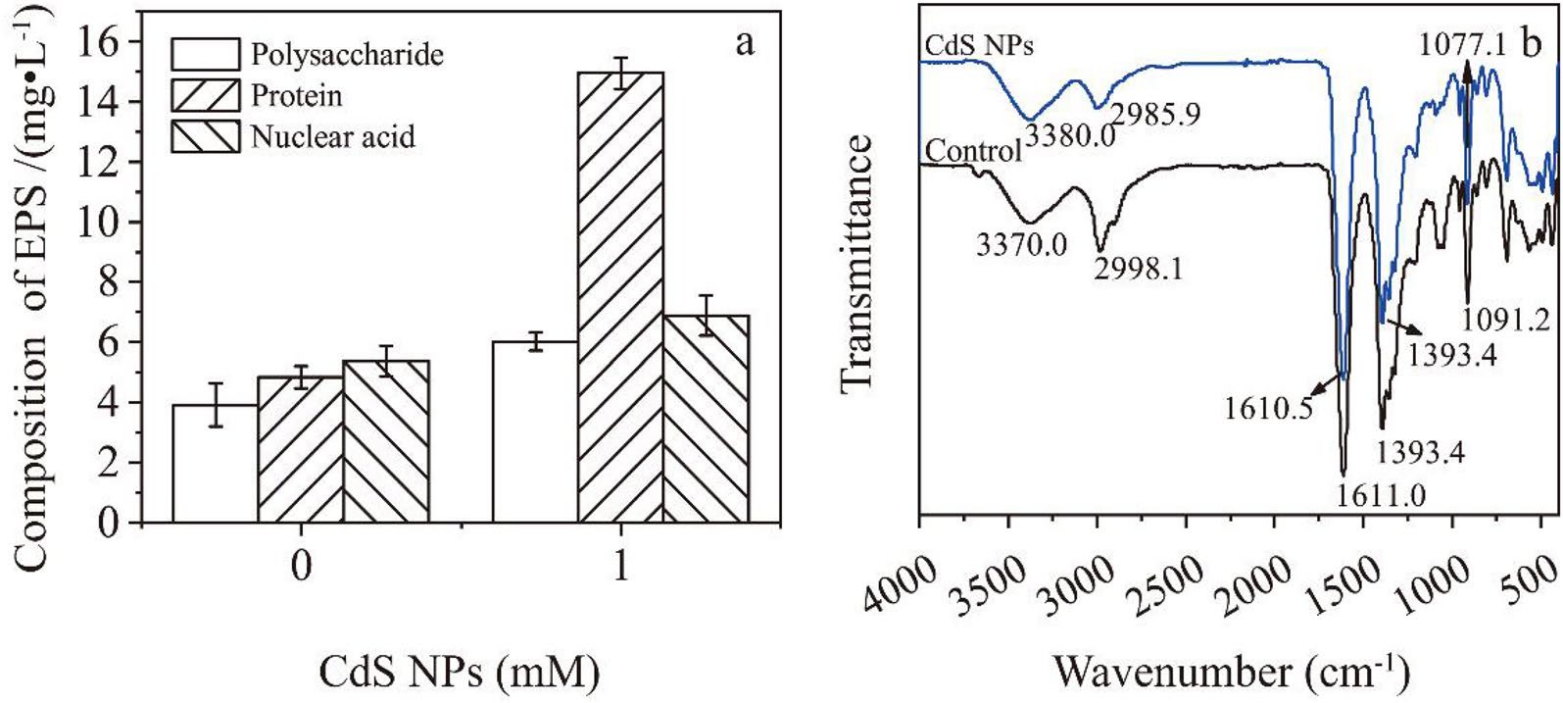
ATR-IR analysis and composition of EPS.** a** Composition of EPS;** b** ATR-IR of EPS

The effect of CdS NPs on EPS synthesis of C09 strain under the condition of 8.1 mW·cm^−2^ light intensity was studied. Compared with the control group, the polysaccharides, proteins, and nucleic acids in 1 mM CdS NPs group increased by 142.5%, 208.9%, and 64.0%, respectively. Among them, the increase of protein was the most obvious among all experimental groups (Fig. 5a). Previous research results showed that the addition of conductive nanomaterials could promote the expression of redox-active proteins, such as cytochromes (Jing et al. 2017). The carbon nanotubes were firstly used to bind the active center of horseradish peroxidase. And then the EET process was observed (Ren et al. 2012). On the limited cell surface area, the higher the protein content in EPS, the more likely it is to facilitate the electron transfer between cells and CdS NPs. It was possible to realize EET between CdS NPs and microorganisms by using CdS NPs as electron donors to transfer electrons to electroactive proteins (Xiao et al. 2017; Li et al. 2016). Therefore, the increased proteins content in EPS of C09 strain might belong to EET related proteins.

In this study, the changes of EPS of C09 strain were analyzed by ATR-IR with/without CdS NPs with results shown in Fig. 5b. The N–H and C=O absorption peaks in proteins were appeared at 3370.0 cm^−1^ and 1611.0 cm^−1^, respectively (Sun et al. 2012). The absorption peak of C-H was appeared at 2998.1 cm^−1^. The absorption peak of -COOH in esters was at 1393.4 cm^−1^. The absorption peak of C–O–C in polysaccharides was at 1091.2 cm^−1^ (Gómez-Ordóñez et al. 2011). There were no obvious shift between the peaks at 3370.0 cm^−1^ and 1611.0 cm^−1^ with/without CdS NPs, indicating that CdS NPs did not affect the protein structure in EPS.

In contrast, there were obviously shifted between the peaks at 2998.1 cm^−1^ and 1091.2 cm^−1^ with/without CdS NPs, indicating that polysaccharides could interact with CdS NPs. It was found that polysaccharides in EPS could adsorb SiO_2_ and ZnO NPs through FITR (Wang et al. 2016). Thus, the ability of CdS NPs to attach to the surface of bacteria was probably related to the polysaccharides in EPS.

**Fig. 6.**
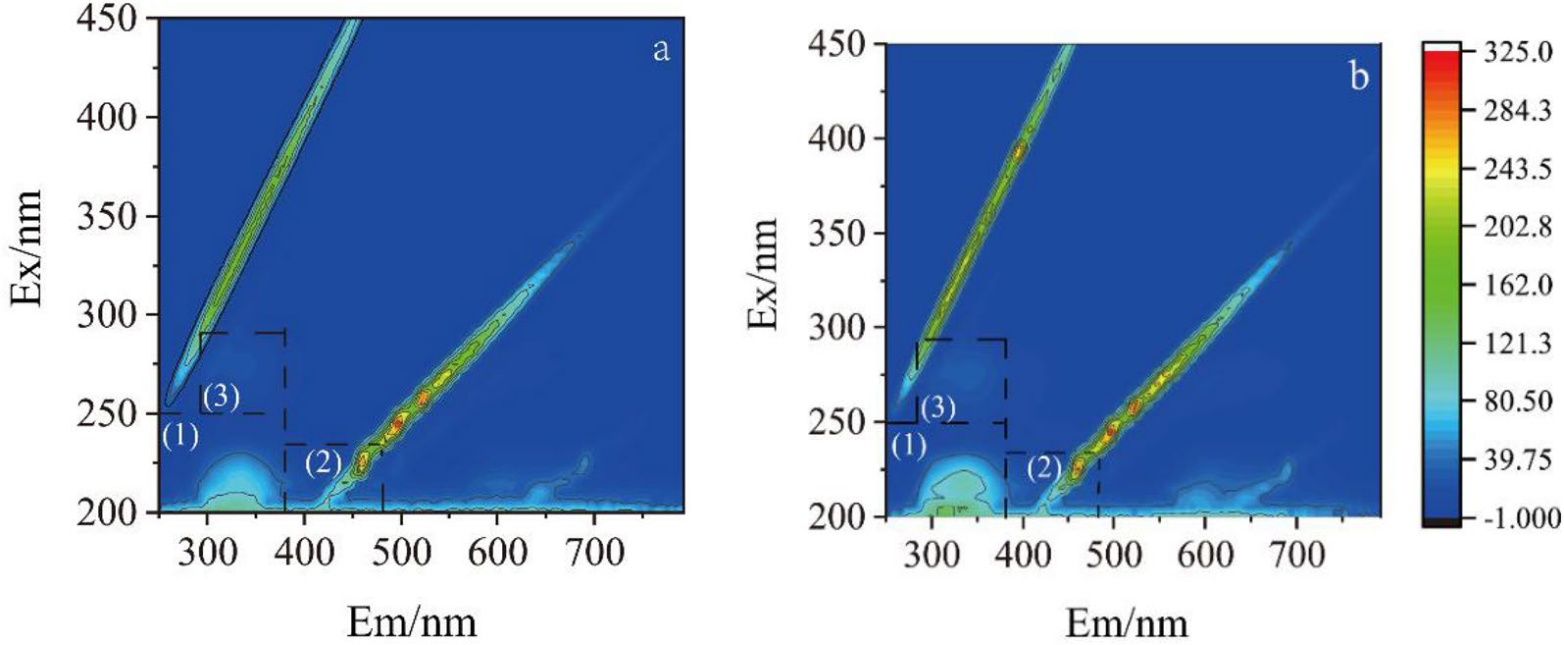
3D-EEM of extracellular polymeric substance.** a** without CdS NPs;** b** with CdS NPs

The EPS composition analysis with/without CdS NPs were carried out by three-dimensional fluorescence spectroscopy with results shown in Fig. 6a and b. Ex/Em 200–250/250–380 nm peaks in region (1) were tyrosine proteins. Ex/Em 200–230/380–480 nm peaks in region (2) were HA (Li et al. 2016). Ex/Em 250–290/290–400 nm peaks in region (3) were soluble microbial metabolites. The signal intensities of the CdS NPs-free and CdS NPs groups increased by 122.2%, 9.0% and 27.7% in regions (1), (2) and (3), respectively, indicating that the content of protein, HA, and the soluble metabolites in EPS could be increased by CdS NPs.

### The effect of extracellular polymeric substances components on sulfate reduction and photo-current

**Fig. 7:**
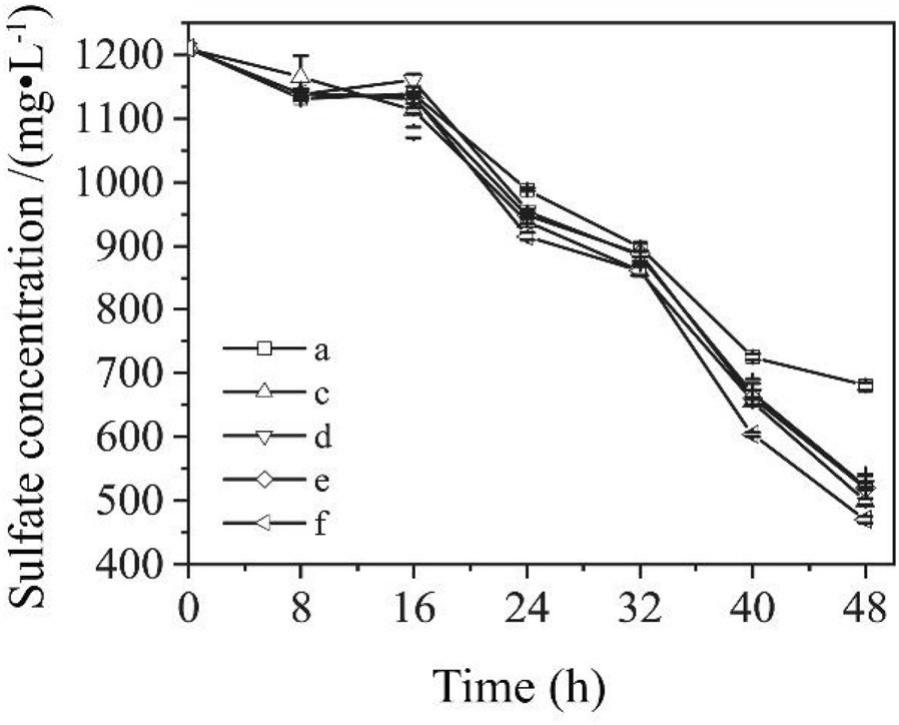
Effects of EPS components on the efficiency of sulfate reduction of C09.** a** Rude EPS and CdS NPs;** b** without EPS, but with CdS NPs;** c** EPS with proteinase treatment;** d** EPS with polysaccharidase treatment;** e** EPS with both proteinase and polyaccharidase;** f** EPS with CdS NPs

The purified EPS was treated with proteinase K, polysaccharidase, and the two enzymes mixtures to investigate the effects of key components of EPS on the sulfate bioreduction process, respectively. The results are shown in Fig. 7.

EPS often contained a variety of components, usually including polysaccharides, proteins, nucleic acids, and HA. These substances had redox properties or electrical activity, and could carry out electron transfer inside and outside the cell. The effect of different components of EPS on sulfate bioreduction process was investigated. The protein and nucleic acid in EPS can be digested by proteinase K and polysaccharase. The sulfate reduction efficiencies of C09 strain of the proteinase K group, the polysaccharase group, the combined proteinase and polysaccharase group and the no enzyme group were 58.8%, 56.8%, 57.1% and 61.2%, respectively. The results showed that the experimental group without any enzymes had the best sulfate reduction efficiency, and the addition of enzymes would decrease the sulfate reduction efficiency. Among them, the polysaccharide in the EPS component could significantly promote the reduction of sulfate. It was consistent with the research results (Yan et al. 2020).

**Fig. 8:**
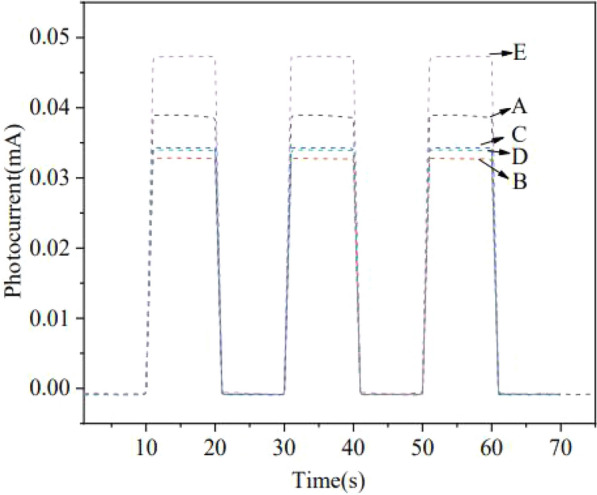
Effect of EPS components on instantaneous photo-current intensity.** A** EPS with proteinase treatment;** B** EPS with both proteinase and polysaccharidase; **C** no-added EPS;** D** EPS with polysaccharidase;** E** added EPS

The transient photo-current intensity was measured to investigate the influence of the composition of EPS on the EET capacity with results shown in Fig. 8.

The results of the instantaneous photo-current produced by CdS NPs showed that the photo-current intensity of both the group of added EPS and the group of EPS with proteinase K treatment were stronger than those of groups with polysaccharidase alone, combination of polysaccharidase and proteinase K, and no EPS. It was demonstrated that externally added EPS had greater photo-current intensity than that without EPS (Zhu et al. 2018). Some components of extracellular EPS, especially polysaccharide, contributed to the separation of electrons from holes and thus might be mainly responsible for the enhanced electron utilization and increased sulfate reduction efficiency of C09 strain (Yan et al. 2020). After the removal of EPS, CdS NPs cannot be adsorbed on the surface of C09 strain, so the electrons generated by CdS NPs could not be used by C09 strain to promote the reduction of sulfate and bacterial growth process.

It was found that the polysaccharide components of EPS might be the electron sacrificial agents to help the separation of electrons and holes of CdS NPs, thereby enhancing the utilization efficiency of electrons by C09 strain.

### Effects of cadmium sulfide nanoparticles on sulfate reduction, biomass, extracellular ·OH, intracellular reactive oxygen species and malondialdehyde content under light conditions

**Fig. 9:**
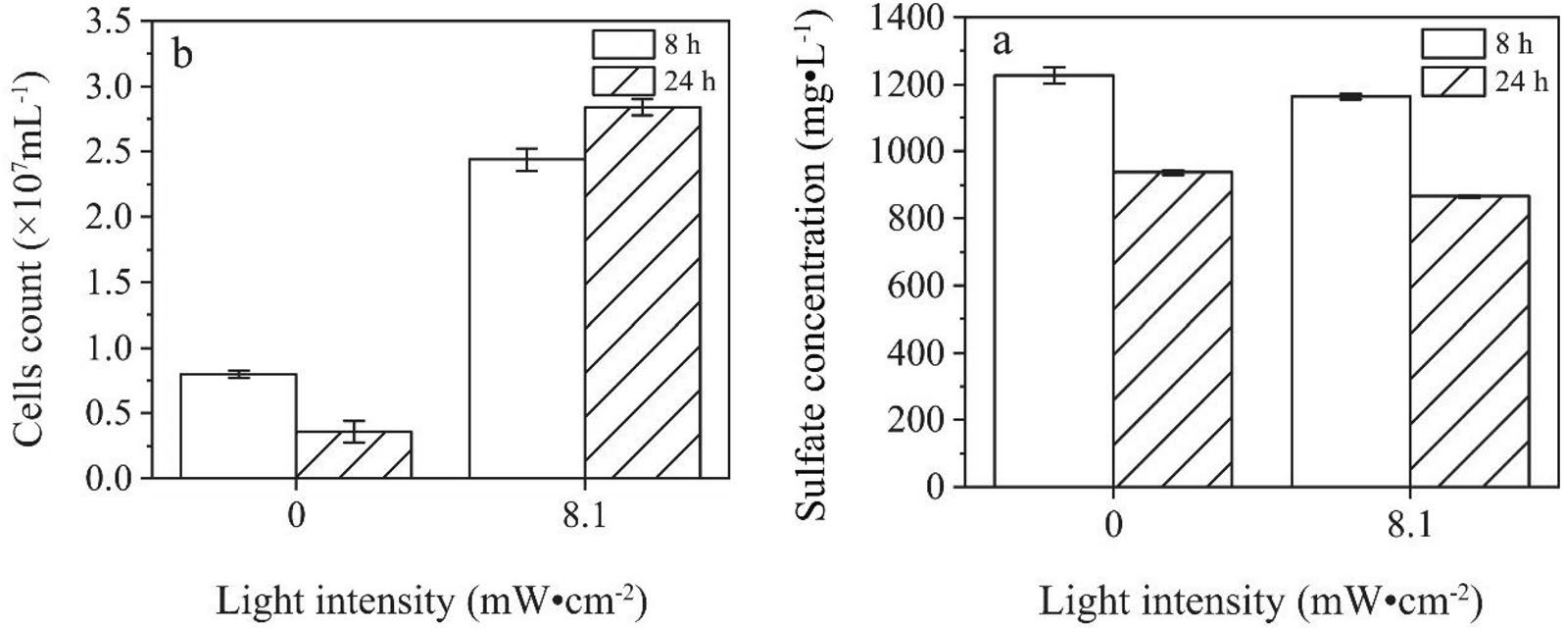
Effect of CdS NPs on growth of C09 and reduction of sulfate under light.** a** Sulfate concentration at 8 h and 24 h;** b** Biomass at 8 h and 24 h

The effects of CdS NPs on the growth of C09 strain and the sulfate reduction process were investigated with results shown in Fig. 3. The sulfate concentration and C09 strain biomass in Fig. 4b were compared and analyzed with results shown in Fig. 9 at 8 h and 24 h.

The sulfate concentration in the 8.1 mW·cm^−2^ group was 7.6% lower than that of the 0 mW·cm^−2^ group (control), and the biomass decreased by 55.0% compared to the control at 8 h, while at 24 h, the biomass increased by 16.4% compared with control. The effect of 5 mg/L of CdS NPs for *E.coli* under the UV irradiation was investigated with results showing that only 3.3% of *E.coli* survived (Shang et al. 2017a, b). However, it was found that CdS NPs could increase the abundance of bacteria under light conditions (Zhu et al. 2018). Therefore, there is no clear conclusion about the influence of nanoparticles on the growth of bacteria.

**Fig. 10:**
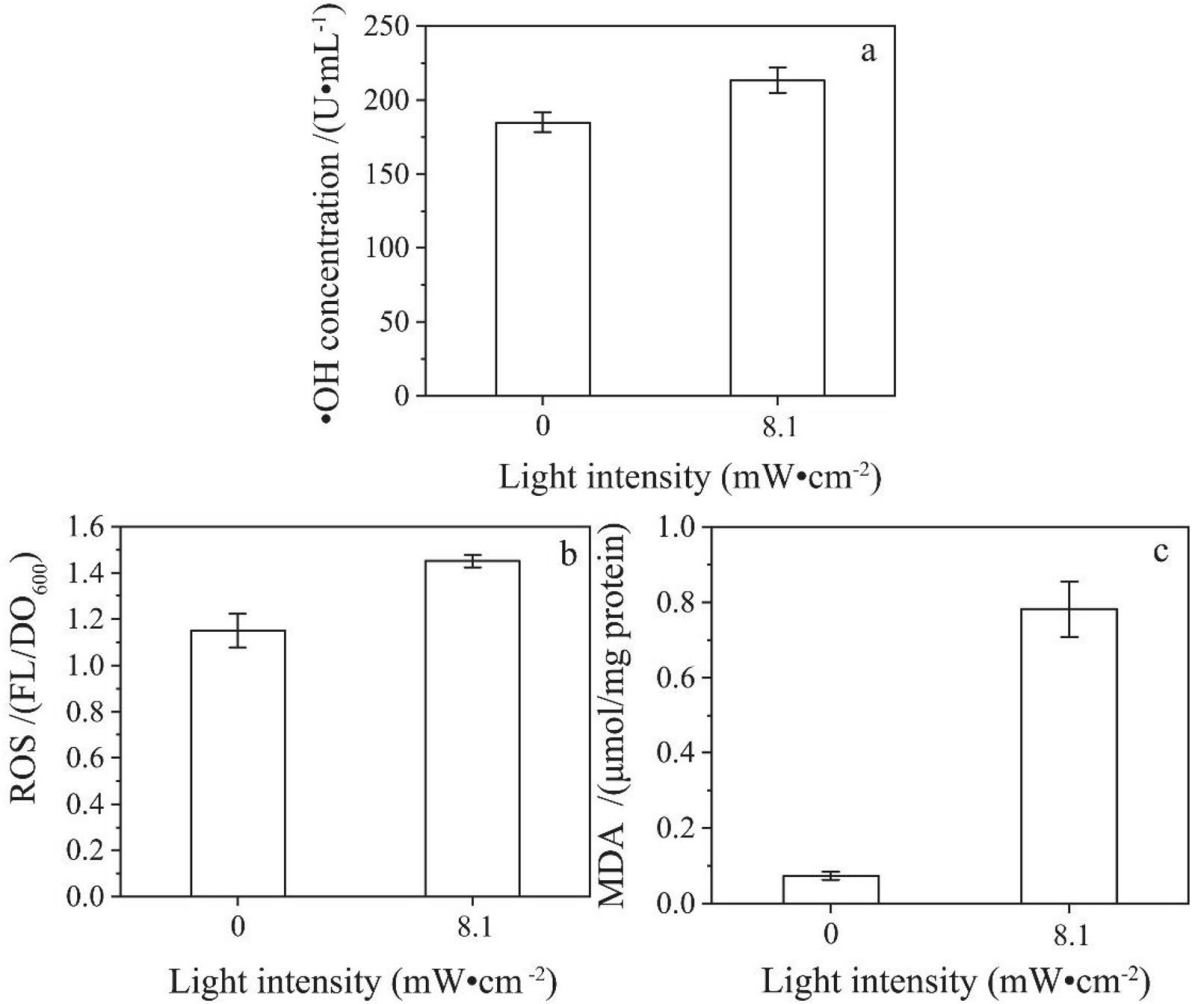
The effects of light intensity on ·OH, intracellular ROS and MDA contents (**A** ·OH concentration;** B** Intracellular ROS content;** C** MDA concentration)

However, CdS NPs could catalyze water to produce ·OH under anaerobic and light conditions. It was found that ·OH could oxidize proteins and the cell membrane lipids (Dutta et al. 2012). Therefore, the oxidation products of cell membranes are used as important indicators for ·OH oxidative damage. In view of the characteristics embodied in the early stage of culture, the extracellular ·OH and intracellular ROS in the early stage of culture were measured. In the experiment, 1 mM CdS NPs were added to the culture medium of C09 strain at light intensity of 0 mW·cm^−2^ (Control) and 8.1 mW·cm^−2^ under the anaerobic condition for 8 h. The extracellular ·OH, intracellular ROS, and MDA content were determined with results shown in Fig. 10a–c.

The contents of extracellular ·OH, intracellular ROS and MDA in the solution increased by 15.4%, 26.1%, and 967.0%, respectively, compared with the control under 8.1 mW·cm^−2^. It was indicated that the content of extracellular ·OH and intracellular ROS, and the cellular peroxidation could be increased by CdS NPs under the light conditions. ZnO NPs would increase the intracellular ROS content of *E. coli* and *S. aureus* under light conditions (Akhil et al. 2017). It was found that the ROS and MDA contents of A549 cells induced by CeO_2_ NPs had increased (Lin et al. 2006). Therefore, the increase in the content of extracellular ·OH and intracellular ROS could affect the growth and metabolism of C09 strain.

Accordingly, CdS NPs could generate cavitation oxidized water to form ·OH under light conditions, the content of extracellular ·OH increases, oxidizes cell membrane lipids, thereby inhibiting the function of cell membrane. However, in the middle and late stages of culture, the inhibitory effect does not seem to be obvious. It was assumed that oxidation radicals could be consumed by sulfate reduction products.

**Fig. 11:**
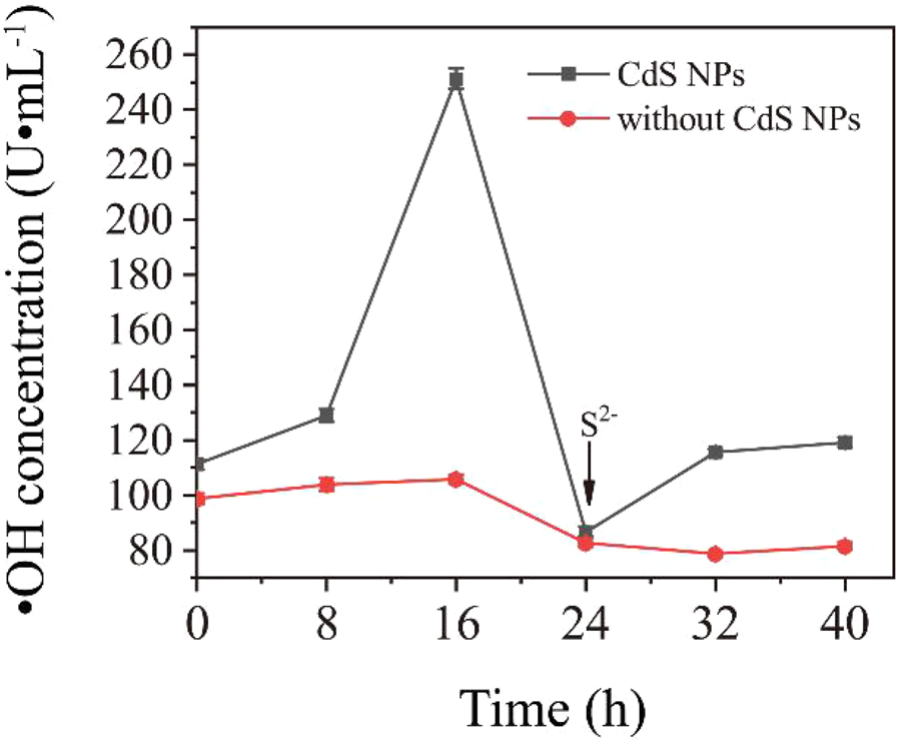
The effect of adding S^2−^ on the content of ·OH in medium

In order to verify the effect of reducing products of this sulfate bioreduction process, such as S^2−^, on the removal of ·OH, a non-biological experiment was designed. At first, the ·OH content in the uninoculated solution were measured every 8 h, and then 100 μL of 0.1 M Na_2_S·9H_2_O solution was added to the medium at 24 h. The experimental results are shown in Fig. 11.

As shown in Fig. 11, the content of ·OH in the solution of the CdS NPs group and the CdS NPs-free group decreased by 65.5% and 21.9% at 24 h, respectively, compared with 16 h, which indicates that S^2−^ could consume the ·OH.

Therefore, the amount of the products of sulfate reduction in the early stage of C09 strain growth was little, and there was a lack of reducing substances to neutralize the ROS produced from CdS NPs. However, a large amount of sulfate was reduced in 24 h to produce more reducing substances, such as S^2−^, HS^−^, H_2_S, which could alleviate the oxidative stress of CdS NPs. Therefore, the oxidative stress produced by CdS NPs in the early growth stage of C09 strain could not be ignored.

However, as an antioxidant, HA is also an important component of EPS. Therefore, it is necessary to investigate the influence of HA on the sulfate bioreduction process.

### Effect of humic acid on the contents of ·OH, reactive oxygen species and malondialdehyde induced by cadmium sulfide nanoparticles

**Fig. 12:**
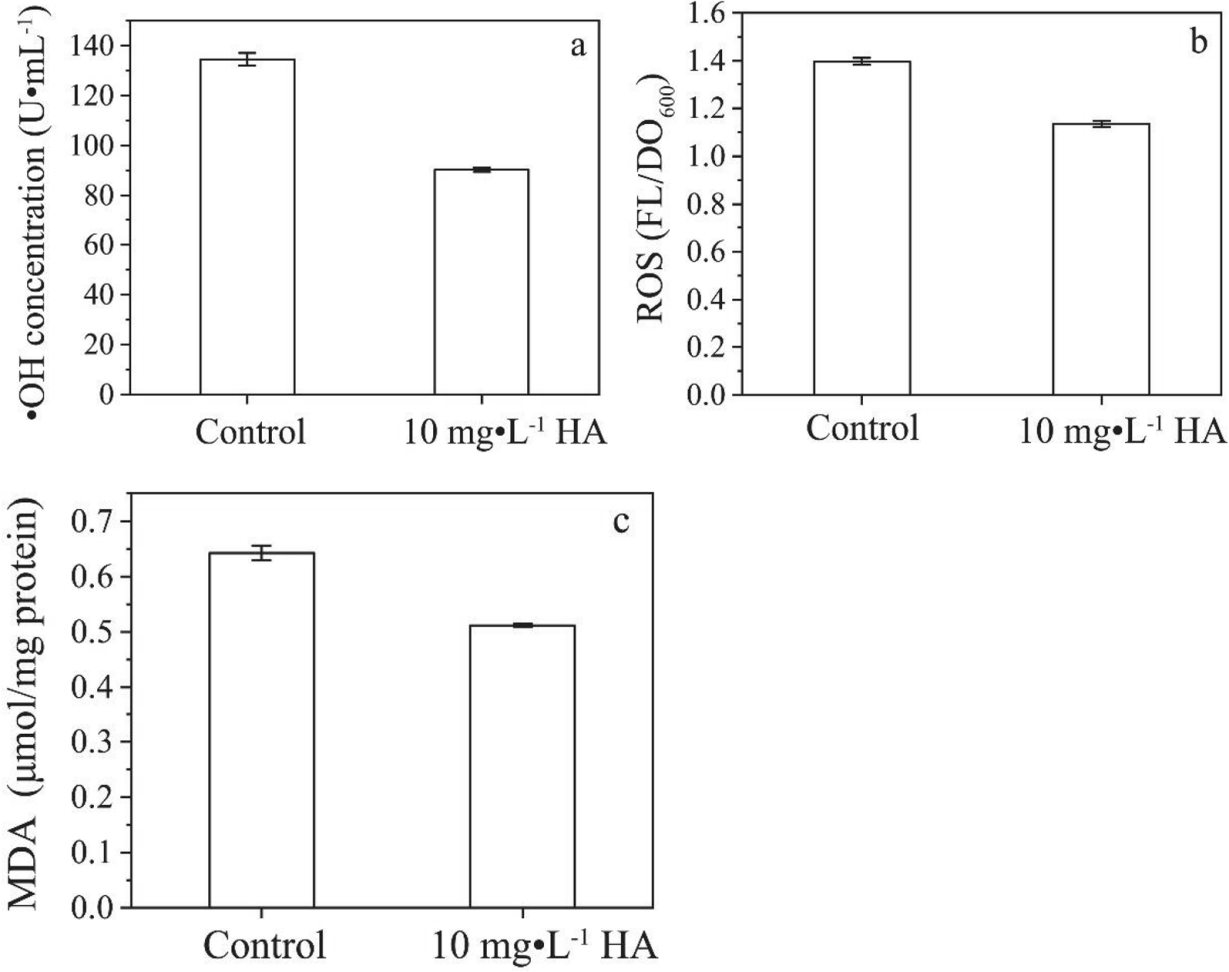
Effects of HA on ·OH, intracellular ROS and MDA contents for C09.** a** ·OH concentration;** b** Intracellular ROS content;** c** MDA concentration

In order to verify that HA could act as an antioxidant, the contents of extracellular ·OH, intracellular ROS and MDA at 8 h of culture were measured after the exogenous addition of HA with results shown in Fig. 12a–c.

It was shown that the content of extracellular ·OH, intracellular ROS and MDA in the experimental group with HA was lower than that of the control. After eight hours of culture, the content of extracellular ·OH, intracellular ROS and MDA in the solution of the HA experimental group was reduced by 32.9%, 18.8%, and 20.5%, respectively, under the light intensity of 8.1 mW·cm^−2^ compared with the control. Thereby the damage of extracellular ·OH and intracellular ROS to cell membranes could be reduced. It was found that the addition of HA could reduce the toxicity of G–CdS (Deng et al. 2016). The reason was that HA could reduce the content of MDA and ROS, increase the activity of CAT and SOD, thereby reducing the oxidative stress induced by G–CdS, and then reduce the damage of lipid, protein and nucleic acid, and even cell death.

In order to solve the problem of oxidative stress induced by CdS NPs in the initial growth phase of C09 strain, one strategy of exogenous addition of HA in the initial growth phase was proposed for this sulfate bioreduction process. It was found that the bioreduction products of sulfate, such as S^2−^, HS^−^ and H_2_S, could effectively alleviate the oxidative stress caused by the photosensitivity of CdS NPs on C09 strain at the middle and later stage of culture. Previous studies had shown that there also existed some HA in EPS of C09 strain, which could reduce the content of MDA and ROS. Therefore, the problem could be solved by exogenous addition of HA in the initial growth phase of C09 strain. This might because that HA could adsorb nanoparticles to reduce their light absorption characteristics (Akhil et al. 2017). It also might be that HA could act as a natural antioxidant undergoing redox reactions with holes and avoiding the generation of holes (Deng et al. 2016).

**Fig. 13:**
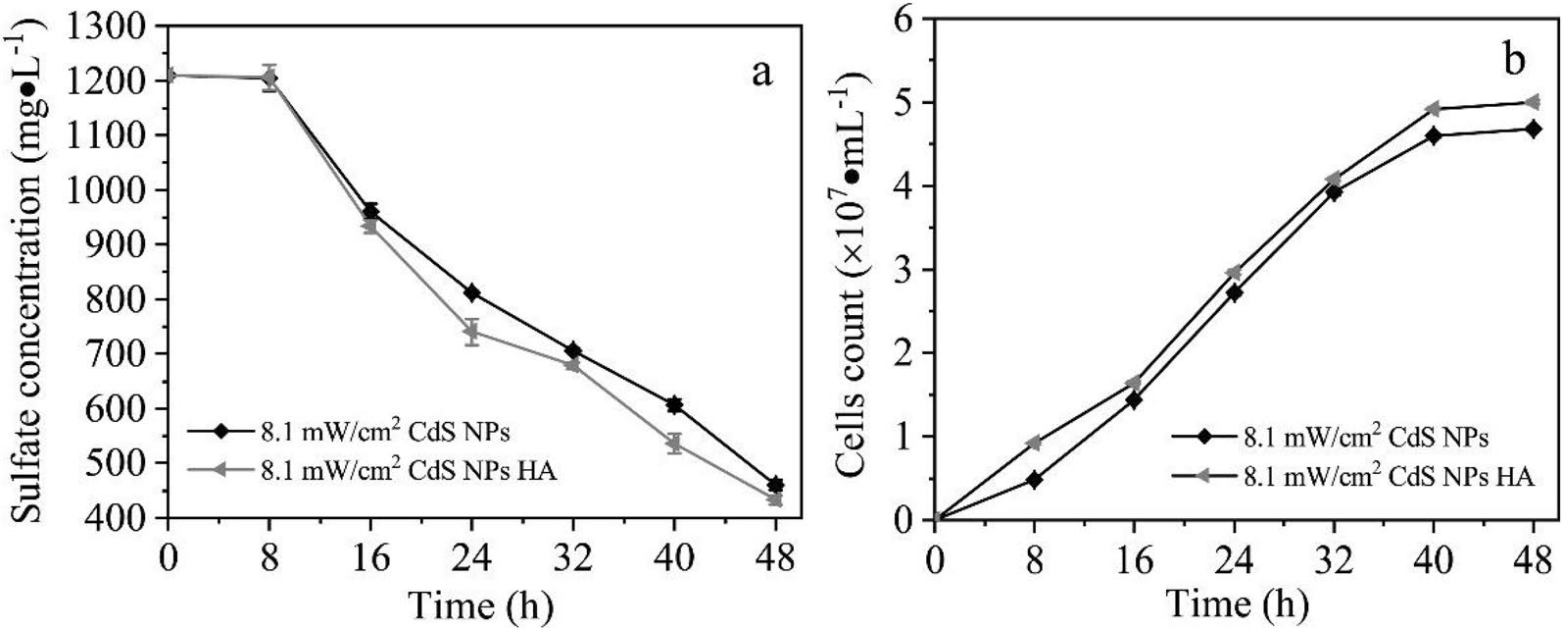
Effects of HA on sulfate reduction and growth of C09.** a** Sulfate concentration;** b** Biomass

In the experiment, the effect of HA on the growth of C09 strain and the process of sulfate reduction was investigated, under the conditions exogenous addition of 10 mg/L of HA at the initial growth phase. The result demonstrated that the sulfate concentration gradually decreased with time, which is shown in Fig. 13a. The exogenous addition of HA at initial growth phase could significantly improve the sulfate reduction efficiency. Among them, the reduction efficiency of sulfate increased by 5.9%. It can also be seen from Fig. 13b that the biomass of C09 strain gradually increased with time. The biomass of C09 strain increased by 6.8% at 8 h after the addition of HA. The bactericidal effect of G–CdS nanocomposites on *E. coli* was studied through the photocatalytic activity of G–CdS nanocomposites (Deng et al. 2016). It was found that the addition of HA through exogenous addition would reduce the toxicity of G–CdS to *E. coli*. In summary, it could be used to alleviate the inhibitory effect of CdS NPs on the initial growth phase of C09 strain by exogenous adding HA or strengthening the biosynthesis ability of HA in EPS, which is of great significance for promoting the reduction of sulfate.

Although CdS NPs have a certain toxicity, the main purpose of this paper was to demonstrate the effect of EET on the growth and metabolism of SRB, and whether the idea was feasible for industrial application. According to the above results, the environment-friendly alternative optical nanomaterials will be developed for the subsequent industrial application process.

### The possible mechanism of C09 strain sulfate reduction strengthened by cadmium sulfide nanoparticles

In summary, a possible mechanism for CdS NPs was proposed to enhance the sulfate reduction process of *D. desulfuricans* C09 strain. Under light conditions, the photoelectrons generated by CdS NPs could pass through the electron receptors (such as cytochrome c, etc.) bound to the extracellular EPS of *D. desulfuricans* C09 strain, and then enter the intracellular electron transport chain to couple with the sulfate reduction process (Kornienko et al. 2016; Deng et al. 2020). The polysaccharide in the extracellular EPS of C09 strain could adsorb CdS NPs on the surface of the bacteria, and it could possibly acts as an electron sacrificial agent to promote the separation of electrons and holes. Therefore, C09 strain gains energy to promote its growth and sulfate reduction efficiency. However, nanoparticles could also generate oxidative free radicals, which had certain negative effects on the initial growth phase of C09 strain, but HA in EPS or exogenous addition HA could eliminate its negative effects. The mechanism of CdS NPs enhancing the sulfate reduction of C09 strain should be further studied to develop more effective and environment-friendly sulfate bioreduction technology.

## Conclusion

A strain with sulfate reduction function was selected from sludge, and identified as *D. desulfuricans* C09. The synthesis of CdS NPs with photosensitivity could increase the efficiency of *D. desulfuricans* C09 to reduce sulfate by 6.4% and significantly increase the biomass under light conditions. CdS NPs could also increase the synthesis and secretion of EPS. Compared with the control group, exogenous addition of EPS and polysaccharides could increase the sulfate reduction efficiency by 3.4% and 5.8%, respectively. Furthermore, it was found that EPS had a significant contribution to the increase of photo-current intensity. The strategy of exogenous addition of HA in the initial growth phase could effectively alleviate the effect of oxidative stress induced by photosensitive nanomaterials CdS NPs.

### Supplementary Information

**Additional file 1. **Screening and identification of sulfate-reducing bacteria.

## Data Availability

All data supporting the findings of this study are available in the article, supporting information, or upon request from the corresponding author.

## Abbreviations

CdS: Cadmium sulfide
EDS: Energy dispersive spectrometer
EEM: Excitation emission matrix
EET: Extracellular electron transfer
EPS: Extracellular polymeric substances
FL: Fluorescence
FT-IR: Fourier transform infrared spectroscopy
G–CdS: Graphene–cadmium sulfide
HA: Humic acid
MDA: Malondialdehyde
NPs: Nanoparticles
PBS: Phosphate balanced solution
ROS: Reactive oxygen species
SEM: Scanning electron microscope
SRB: Sulfate reducing bacteria
TBA: Thiobarbituric acid
XRD: X-ray diffraction
