# Supplementary material for: Effects of cadmium sulfide nanoparticles on sulfate bioreduction and oxidative stress in Desulfovibrio desulfuricans
Source: Bioresour Bioprocess. 2022 Apr 1;9(1):35. doi: 10.1186/s40643-022-00523-5 (PMC10991916; doi:10.1186/s40643-022-00523-5)
Supplement: Supplementary file 1 — Additional file 1. Screening and identification of sulfate-reducing bacteria. [file 40643_2022_523_MOESM1_ESM.pdf]

Supplementary material

1 Screening and identification of sulfate-reducing bacteria

In this study, a sulfate-reducing strain was screened from sludge in our lab. After the primary and second isolation, the partial results of sulfate reduction efficiency of sulfate-reducing bacteria were shown in Fig. S1 of supplementary material. The sulfate reduction efficiency of No. 9 was the highest than those of others. The results of physiological and biochemical characteristics of No. 9 were shown in Table S1 of supplementary material, which were consistent with those of *D. desulfurican*. The results of sequence of 16S rRNA of No. 9 indicated that there were 99.7% homology similarity with that of (NR_104990.1) *D. desulfuricans* strain Essex 6. The phylogenetic tree of No. 9 based on 16S rRNA sequences was shown in Fig. S2 of supplementary material. According to above results, the target strain No. 9 was identified as genus *Desulfovibrio* and named as *D. desulfuricans* C09.

Table S1. Results of the physiological and biochemical experiment of *Desulfovibrio desulfuricans* C09

Test name	Results
Gram staining	-
Indole test	+
Methyl red test	-
V-P	-
Gelatin	-
Hydrogen sulfide	+

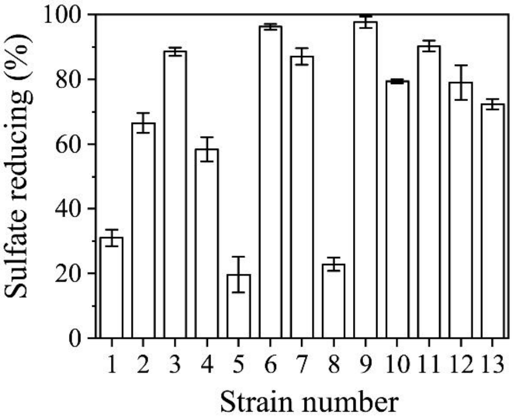

Fig. S1. Sulfate reduction efficiency of part of isolated strains

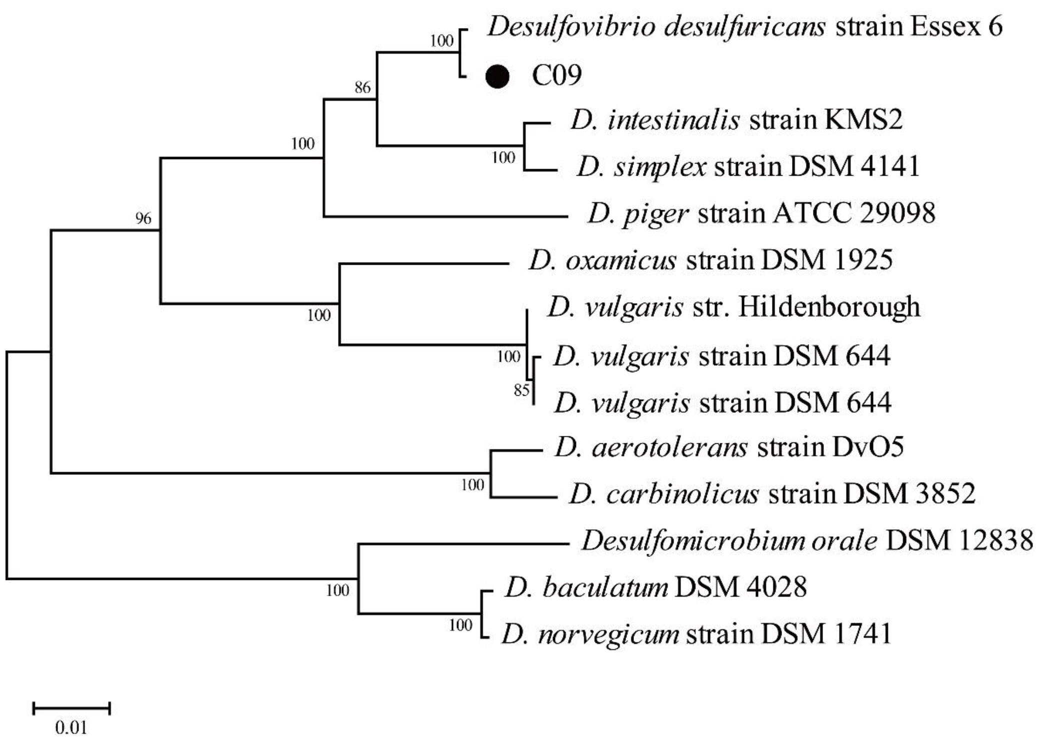

Fig. S2. Phylogenetic tree of C09 based on 16S rRNA gene sequences

The gene sequence of C09 strain

AAGTCGACGCGAAAGGGACTTCGGTCCTGAGTAAAGTGGCGCACGGGTGAGTAACGCGTGGATAATCTGCCCTTATGATCGGGATAACAGTTGGAAACGGCTGCTAATACCGGATACGCTCAAAATGAACTTTTTGAGGAAAGATGGCCTCTGCTTGCATGCTATCACGTAAGGATGAGTCCGCGTCCCATTAGCTTGTTGGCGGGGTAACGGCCCACCAAGGCAACGATGGGTAGCCGATTTGAGAGGATGATCGGCCACACTGGAACTGAAACACGGTCCAGACTCCTACGGGAGGCAGCAGTGGGGAATATTGCGCAATGGGCGAAAGCCTGACGCAGCGACGCCGCGTGAGGGATGAAGGTTTTCGGATCGTAAACCTCTGTCAGAAGGGAAGAAACTACGTTGTGCTAATCAGCAGCGTACTGACGGTACCTTCAAAGGAAGCACCGGCTAACTCCGTGCCAGCAGCCGCGGTAATACGGAGGGTGCAAGCGTTAATCGGAATTACTGGGCGTAAAGCGCACGTAGGCTGTAGTGTAAGTCAGGGGTGAAATCCCACGGCTCAACCGTGGAACTGCCTTTGATACTGCACAACTTGAATCCGGGAGAGGGTGGCGGAATTCCAGGTGTAGGAGTGAAATCCGTAGATATCTGGAGGAACATCAGTGGCGAAGGCGGCCACCTGGACCGGTATTGACGCTGAGGTGCGAAAGCGTGGGGAGCAAACAGGATTAGATACCCTGGTAGTCCACGCTGTAAACGATGGATGCTAGATGTCGGGGAGTATTCTTCGGTGTCGTAGTTAACGCGTTAAGCATCCCGCCTGGGGAGTACGGTCGCAAGGCTGAAACTCAAAGAAATTGACGGGGGCCCGCACAAGCGGTGGAGTATGTGGTTTAATTCGATGCAACGCGAAGAACCTTACCTAGGTTTGACATCCACGGAACCCTCCCGAAAGGAGGGGTGCCCTTCGGGGAGCCGTGTGACAGGTGCTGCATGGCTGTCGTCAGCTCGTGTCGTGAGATGTTGGGTTAAGTCCCGCAACGAGCGCAACCCCTATGGATAGTTGCCAGCAAGTAATGTTGGGCACTCTATTCAGACTGCCCGGGTTAACCGGGAGGAAGGTGGGGACGACGTCAAGTCATCATGGCCCTTACGCCTAGGGCTACACACGTACTACAATGGCGCGCACAAAGGGGAGCGAGACCGCGAGGTGGAGCCAATCCCAAAAAACGCGTCCCAGTCCGGATTGCAGTCTGCAACTCGACTGCATGAAGTTGGAATCGCTAGTAATTCGAGATCAGCATGCTCGGGTGAATGCGTTCCCGGGCCTTGTACACACCGCCCGTCACACCACGAAAGTCGGTTTTACCCGAAGCCGGTGAGCCAACCAGCAATGGAGGC
